# Mapping the therapeutic landscape of CRISPR-Cas9 for combating age-related diseases

**DOI:** 10.3389/fgeed.2025.1558432

**Published:** 2025-04-04

**Authors:** Qiyu He, Yida Wang, Zhimin Tan, Xian Zhang, Chao Yu, Xiaoqin Jiang

**Affiliations:** ^1^ Department of Urology, West China Hospital of Sichuan University, Chengdu, Sichuan, China; ^2^ Key Laboratory of BioResource and Eco-Environment of Ministry of Education, College of Life Science, Sichuan University, Chengdu, Sichuan, China; ^3^ Department of Anesthesiology, West China Hospital of Sichuan University, Chengdu, Sichuan, China; ^4^ Department of Anesthesiology, West China Second Hospital of Sichuan University, Chengdu, Sichuan, China; ^5^ Key Laboratory of Birth Defects and Related Diseases of Women and Children, Sichuan University, Ministry of Education, Chengdu, China; ^6^ Department of Anesthesiology, Chengdu Hi-Tech Zone Hospital for Women and Children, Chengdu, China

**Keywords:** age-related diseases, bibliometric analysis, CRISPR-Cas9, genome editing, gene therapy

## Abstract

CRISPR-Cas9 (clustered regularly interspaced short palindromic repeats-associated protein 9) has emerged as a transformative genome-editing tool with significant therapeutic potential for age-related diseases, including Alzheimer’s disease, Parkinson’s disease, cardiovascular disorders, and osteoporosis. This study presents a bibliometric analysis of CRISPR-Cas9 research in age-related diseases, identifying key contributors, major research hotspots, and critical technological advancements. While promising applications have been demonstrated in gene repair, functional regulation, and molecular interventions, significant barriers persist, including off-target effects, low delivery efficiency, and limited editing in non-dividing cells. Ethical concerns over germline editing and gaps in long-term safety data further complicate clinical translation. Future directions emphasize the development of high-precision Cas9 variants, homology-directed repair-independent tools, and efficient delivery systems, alongside the establishment of international regulatory frameworks and multicenter clinical trials. These efforts are essential to fully realize the potential of CRISPR-Cas9 in addressing the global health challenges of aging.

## 1 Introduction

The global aging population has led to a sharp rise in age-related diseases, including Alzheimer’s (Hou et al., 2019), Parkinson’s (Lopes et al., 2022), cardiovascular disorders (Liang et al., 2024a), and cancer (Berben et al., 2021), placing considerable strain on healthcare systems due to their high prevalence and prolonged care requirements. Aging is a multifaceted biological process shaped by genetic (Maldonado et al., 2023), epigenetic (Liang et al., 2024b), and environmental factors (Dodig et al., 2019), characterized by key hallmarks such as genomic instability, aberrant epigenetic regulation, telomere attrition, mitochondrial dysfunction, and chronic low-grade inflammation (López-Otín et al., 2023).

**FIGURE 1:**
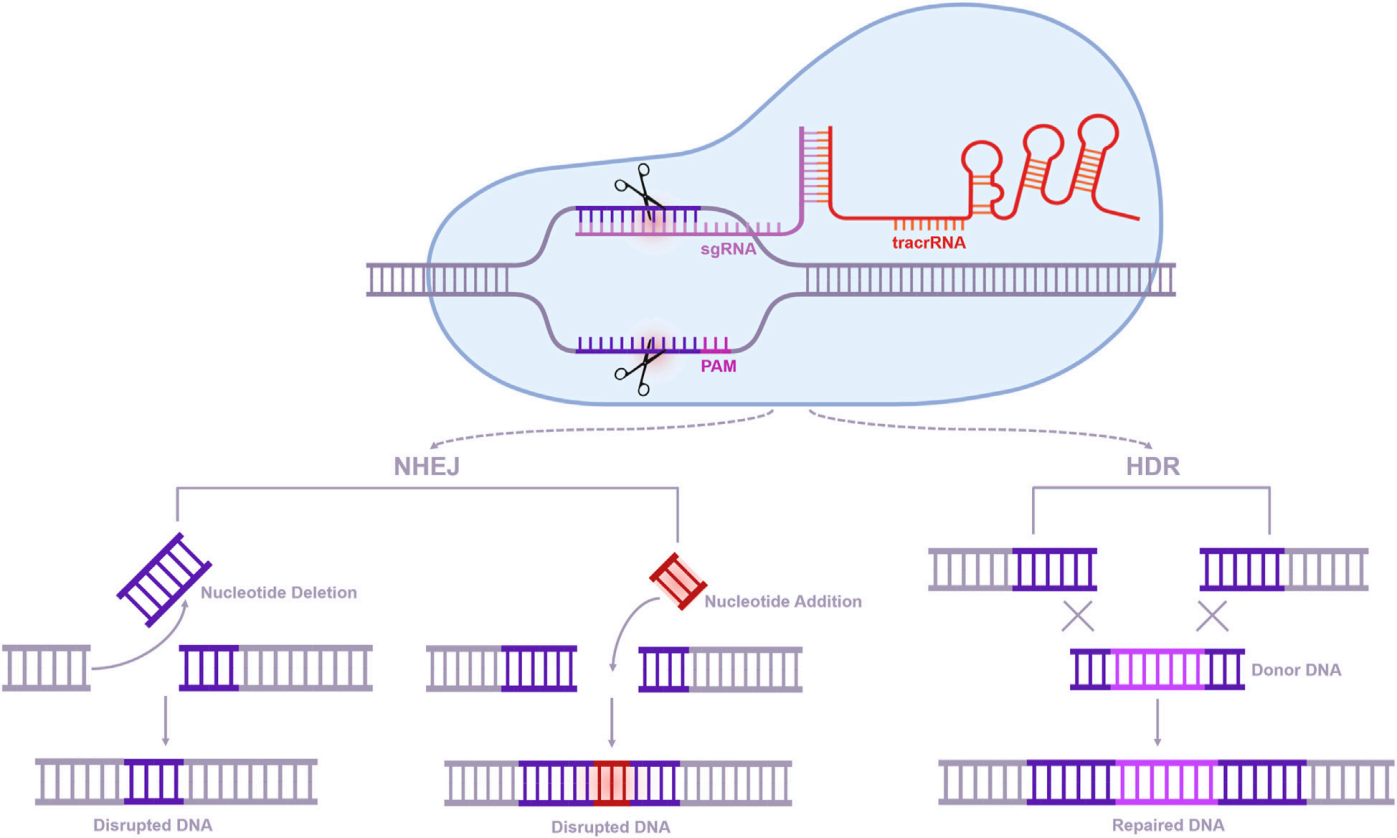
CRISPR-Cas9 system mechanism and DNA repair pathways.

CRISPR-Cas9 (clustered regularly interspaced short palindromic repeats-associated protein 9), a transformative genome-editing tool (Figure 1), enables precise targeting of genes and epigenetic regulators implicated in aging (Doudna and Charpentier, 2014). It has demonstrated potential in mitigating cellular senescence, genomic instability, and inflammatory processes (Hazrati et al., 2022). Applications in age-related diseases include correcting pathogenic mutations in Alzheimer’s and Parkinson’s, modulating metabolic pathways in cardiovascular disorders, and slowing the progression of osteoporosis and metabolic dysfunctions (Caobi et al., 2020). With superior precision and efficiency compared to conventional methods, CRISPR-Cas9 represents a powerful approach to unraveling and addressing the molecular mechanisms of aging (Zhang et al., 2024).

Despite significant advancements, research on the therapeutic applications of CRISPR-Cas9 in age-related diseases remains fragmented, with limited understanding of its mechanistic roles, long-term effects, and safety profiles (Caobi et al., 2020). Clinical translation faces major hurdles, including off-target effects leading to genomic instability, low tissue-specific delivery efficiency, and reduced efficacy in non-dividing cells (Dai et al., 2016). Ethical concerns surrounding germline editing and uncertainties about long-term impacts further constrain widespread adoption (Cwik, 2020). Bibliometric analysis offers a valuable framework to evaluate global research trends, identify key contributors, and map critical gaps, providing essential insights to guide future research and advance the field (Moed, 2009).

This study aims to construct a comprehensive landscape of CRISPR-Cas9’s therapeutic applications in age-related diseases, highlighting current progress, technological advancements, and translational barriers. Through the integration of bibliometric analysis and an evaluation of its therapeutic potential and challenges, this work offers insights to accelerate the clinical translation of CRISPR-Cas9 in addressing the health challenges of aging.

## 2 Materials and methods

### 2.1 Data sources and retrieval strategies

This study systematically retrieved CRISPR-Cas9 research related to age-associated diseases from PubMed, Embase, Cochrane Library, Scopus, and Web of Science, covering the period from January 1, 2005, to December 31, 2024. To ensure consistency and reliability, two authors independently conducted searches on the same day. Detailed search strategies and database-specific results are provided in Supplementary Table S1.

**FIGURE 2:**
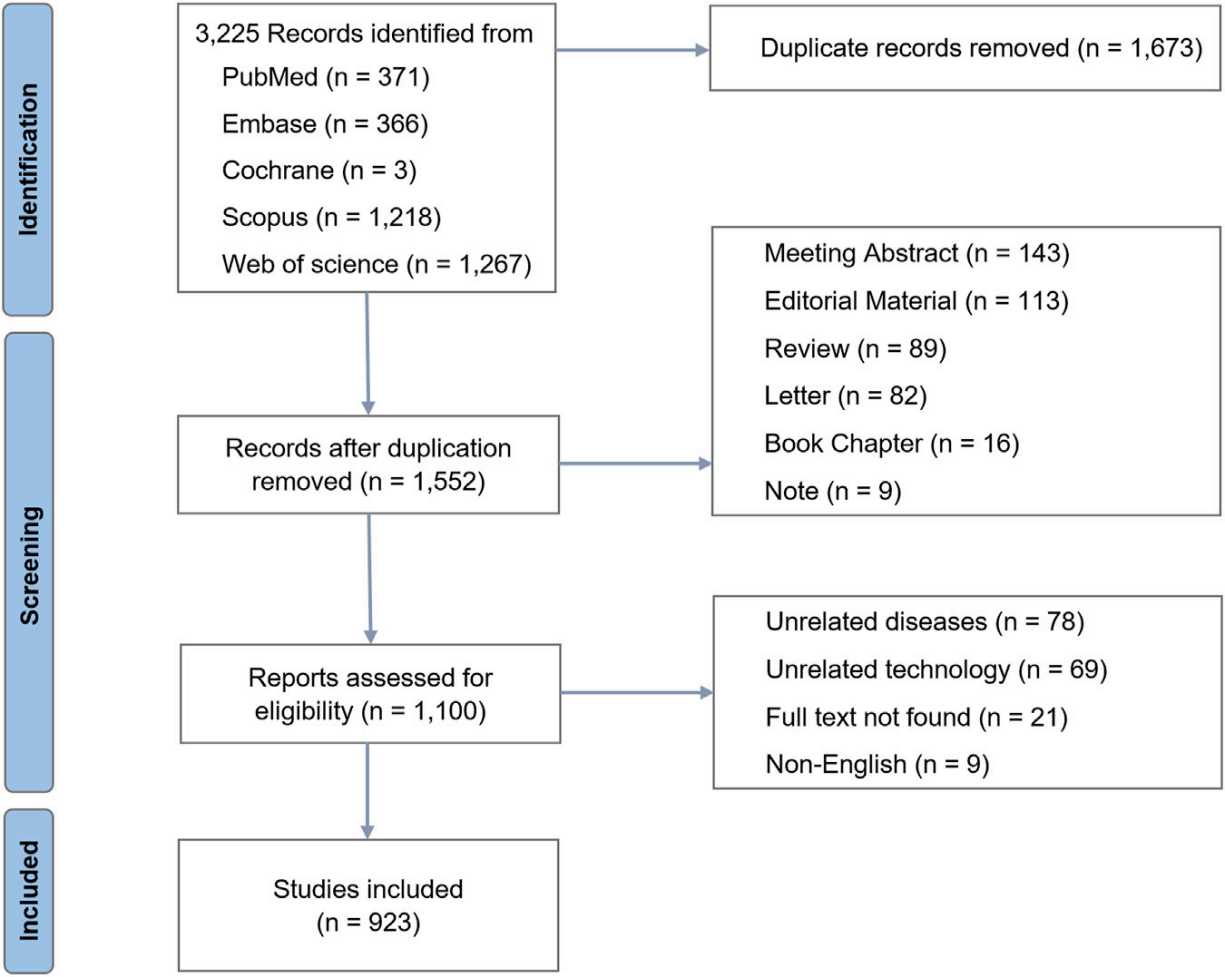
Flowchart of study selection for CRISPR-Cas9 research in age-related diseases.

The inclusion criteria were as follows: 1) studies investigating CRISPR-Cas9 interventions targeting molecular pathways involved in age-associated diseases; 2) experimental validation through *in vitro*, *in vivo*, or clinical studies; and 3) provision of primary data analysis. Exclusion criteria included: 1) duplicate publications; 2) meeting abstracts; 3) editorial materials; 4) letters; 5) book chapters; 6) notes; 7) studies on unrelated diseases; 8) studies involving unrelated technologies; 9) articles with unavailable full texts; and 10) non-English language articles. Title, abstract and full-text screenings were independently conducted by two authors using pre-specified eligibility criteria and a piloted data extraction form. Discrepancies were resolved through iterative discussion, and unresolved conflicts were adjudicated by a third reviewer according to predefined rules, which included: 1) focusing on age-associated diseases as classified by the WHO; 2) mandatory re-examination of full texts for studies with ambiguous CRISPR-Cas9 applications; and 3) documenting all decisions with cross-references to the eligibility criteria. Ultimately, 923 original articles met the inclusion criteria and were included in the final analysis (Figure 2).

### 2.2 Data acquisition and analysis

Bibliometric information, including publication and citation counts, countries, institutions, authors, journals, co-cited references, funding agencies, and keywords, was exported for analysis. Following bibliometric principles, co-occurrence analysis of countries, authors, institutions, and co-cited references was performed using CiteSpace to create visual atlases (Donthu et al., 2021). CiteSpace also facilitated cluster analysis and keyword burst detection. Cluster analysis, an unsupervised learning algorithm widely applied in machine learning and information recognition, was employed to uncover patterns and groupings within the data (Shirkhorshidi et al., 2014). A global distribution and collaboration map of CRISPR-Cas9 research in age-related diseases was generated using Python (v3.10.12), with Geopandas (v0.13.2) for geospatial data processing and Matplotlib (v3.7.1) for visualization. Additionally, the publication growth trend was modeled in Excel through a fitting formula, offering insights into research trajectories.

## 3 Results

### 3.1 Annual publication and citation trend analysis

**FIGURE 3:**
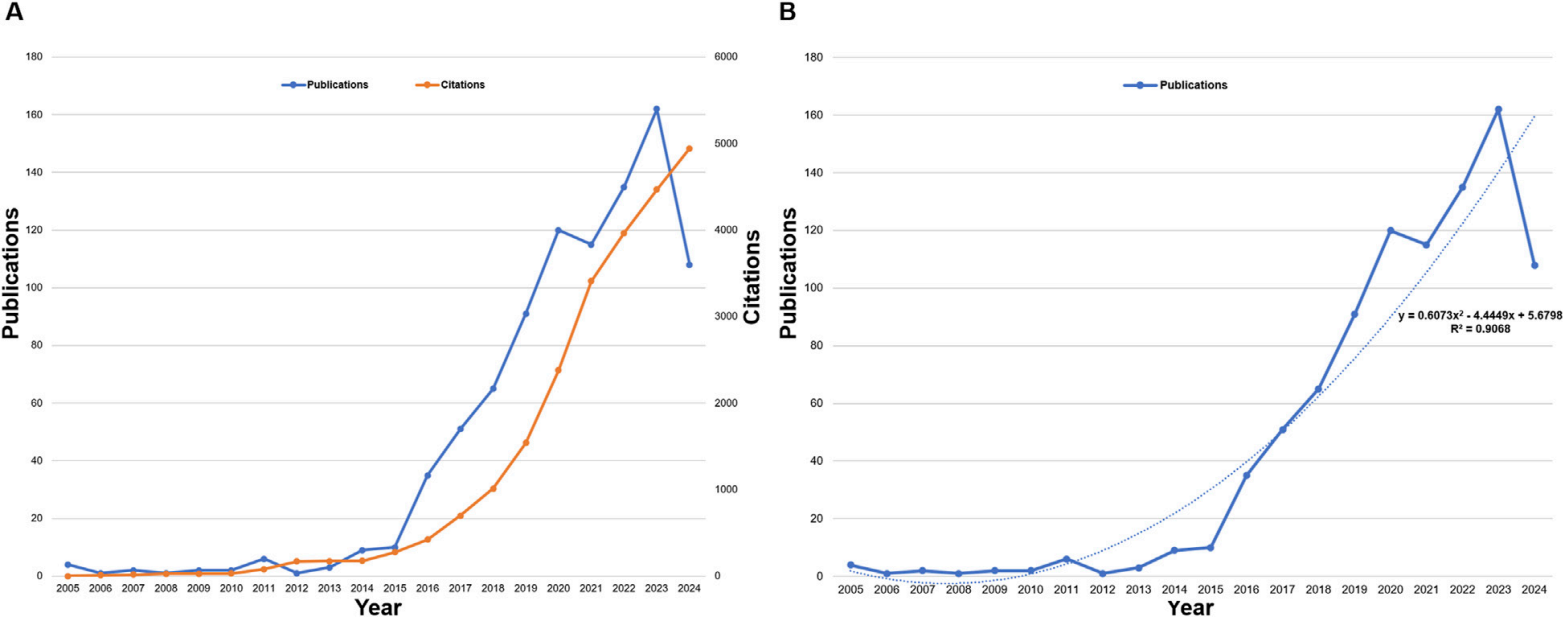
**(A)** Annual trends in publications and citations, and **(B)** curve fitting of annual growth trends in publications on CRISPR-Cas9 research.

The growth of annual publications and citations reflects the dynamic expansion of the field. Total citations have surged to 23,835 (Figure 3A), with an average of 26.03 citations per article. The H-index, a robust metric of research impact, stands at 73, signifying that 73 articles have been cited at least 73 times (Lü et al., 2016). To illustrate the relationship between publication year and research output, a fitting formula was applied, where “x” represents the year and “y” denotes the annual publication count (y = 0.6073x^2^ - 4.449x + 5.6798, R^2^ = 0.9068) (Figure 3B). This exponential growth pattern correlates with critical therapeutic milestones in the field. The acceleration phase (2018–2022) coincided with the first CRISPR-based therapies entering clinical trials for age-related conditions, including CTX001 for β-thalassemia and sickle cell disease (Frangoul et al., 2021).

### 3.2 Country and region

**FIGURE 4:**
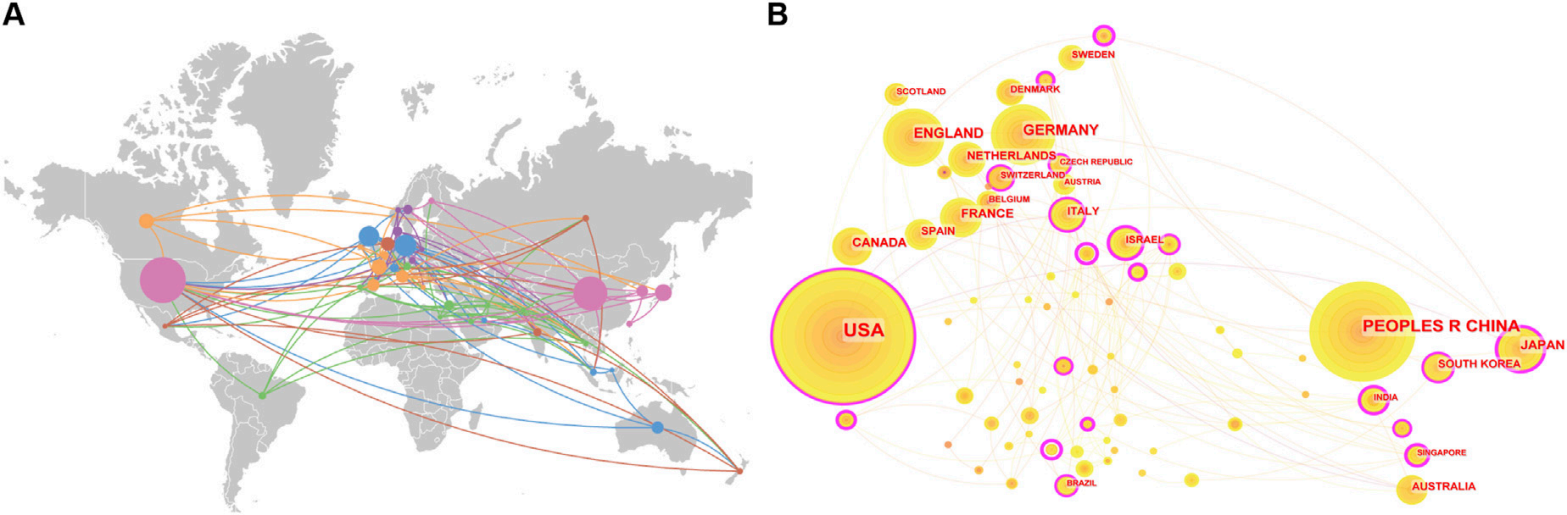
**(A)** Global distribution, and **(B)** country co-occurrence network map of CRISPR-Cas9 research and international collaborations in age-related diseases.

Research on CRISPR-Cas9 in age-related diseases is distributed across 73 countries and regions, with the United States and China dominating the field, contributing 44.78% of the total publications (Figure 4A). Germany, England, and France also feature prominently, as reflected by their significant nodes in the country co-occurrence network, underscoring their substantial influence in this domain (Figure 4B). The international collaboration network comprises 135 interconnections among participating countries, resulting in a network density of 0.051. Although this indicates a relatively well-established framework for global cooperation, opportunities for strengthening cross-border partnerships remain (Figure 4B). These insights underscore both the leadership of key nations and the potential for fostering deeper international engagement.

Our analysis also reveals national differences in CRISPR-Cas9 research on age-related diseases. The United States focuses on aging mechanisms and neurodegenerative disease models (Marschallinger et al., 2020), while China leads in gene screening, particularly whole-genome functional screening, and clinical translation (Li and Huang, 2018). Germany and the United Kingdom emphasize multi-center studies on neurodegenerative diseases (Barazesh et al., 2021), and France pioneers gene-editing therapies for cardiovascular diseases (Vermersch et al., 2020). These variations reflect distinct national strategies for advancing CRISPR-Cas9 in age-related diseases. Future studies could explore the underlying factors, such as demographic needs, funding priorities, and available expertise.

### 3.3 Author and institution

**FIGURE 5:**
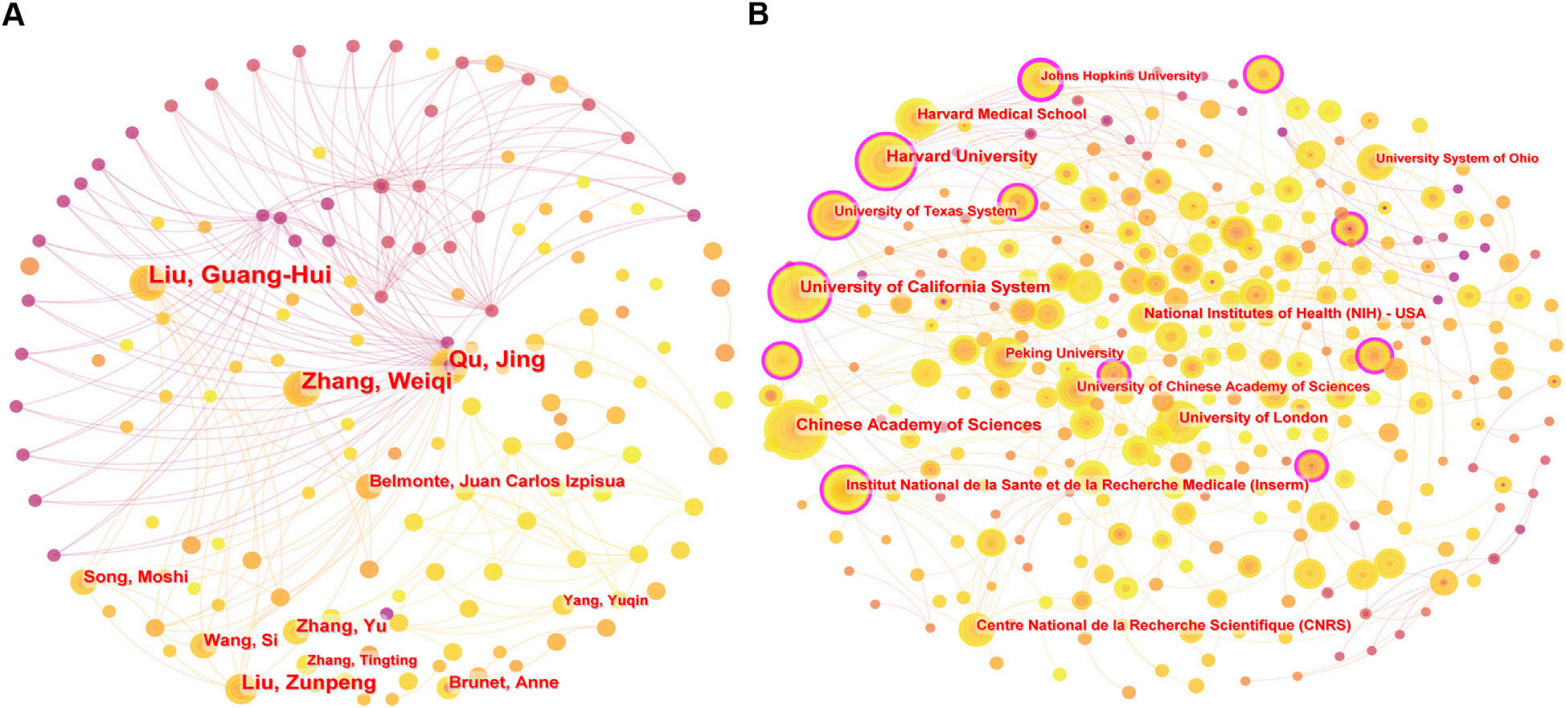
**(A)** Author co-occurrence network map, and **(B)** institution co-occurrence network map in CRISPR-Cas9 research on age-related diseases.

The top ten authors and institutions (Supplementary Tables S2, S3) primarily originate from the United States and China, highlighting the leading role of these two countries in the field. Several collaborative networks have emerged, with strong ties within groups but limited connections between them (Figure 5). Liu Guang-Hui and Qu Jing, both from the Chinese Academy of Sciences, are among the most prolific authors. Other notable contributors include Belmonte Juan Carlos Izpisua (Salk Institute) and Brunet Anne (Stanford University). Harvard University and the Chinese Academy of Sciences are the most productive institutions, with 87 and 77 publications, respectively, followed by the University of California system, Institut National de la Santé et de la Recherche Médicale, and the University of London, which also serve as key hubs for collaboration.

Despite robust partnerships within clusters, intergroup collaboration remains limited, reflecting potential regional or disciplinary barriers. Enhancing these connections could accelerate advancements in CRISPR-Cas9 applications for age-related diseases.

### 3.4 Funding agency

A total of 414 funding agencies were identified. The top ten funding bodies subsidized 399 studies, accounting for 43.23% of the literature in this field. The top three donors were the National Institutes of Health (77), the United States Department of Health and Human Services (57), and the National Natural Science Foundation of China (51). These results indicate that the United States and Chinese governments have provided great support and funding for CRISPR-Cas9 research on age-related diseases (Supplementary Table S4.

### 3.5 Core journal

The top ten journals contributing to CRISPR-Cas9 research on age-related diseases are recognized as core outlets in the field, collectively accounting for 18.42% of total publications (Supplementary Table S5). All are classified within Q1 and Q2 categories of the Journal Citation Reports (2024) and specialize in molecular biology, genetics, and aging research. This reflects the interdisciplinary scope of CRISPR-Cas9 applications in tackling complex biological processes and age-related disorders. Given their prominence and focus, these journals are poised to remain pivotal in disseminating future breakthroughs, continuing to shape progress in this rapidly advancing domain.

Besides, the evolution of publication patterns across these journals tracks the therapeutic maturation of the field. Early publications predominantly appeared in basic science journals focusing on CRISPR mechanisms and tool development. By 2018–2020, a notable shift occurred toward disease-specific journals, coinciding with proof-of-concept studies demonstrating therapeutic efficacy in models of age-related diseases. Most recently (2021-present), clinical and translational journals have featured prominently, reflecting the advancement of CRISPR therapies toward patient applications. This progression through journal categories illustrates the field’s transition from technical innovation to therapeutic implementation, with recent publications increasingly addressing clinical considerations such as delivery optimization, safety profiling, and patient selection criteria.

### 3.6 Co-citation and citation analysis

**FIGURE 6:**
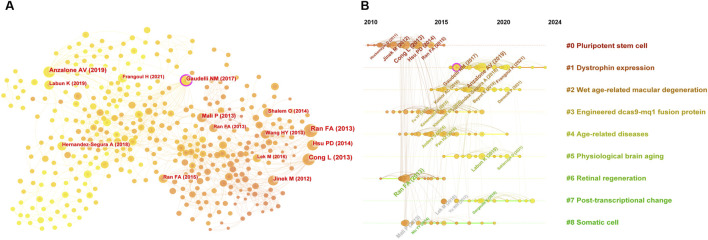
**(A)** Knowledge map of co-citation literature, and **(B)** timeline visualization of co-cited studies on CRISPR-Cas9 for combating age-related diseases.

Co-citation analysis, which identifies studies frequently cited together, provides insights into the intellectual framework of a research field (Small and Griffith, 1974). Using CiteSpace, a co-citation network comprising 716 nodes and 2,216 connections with a topological density of 0.0087 was constructed (Figure 6A), revealing key articles and their interrelationships. A timeline visualization (Figure 6B) illustrates the field’s progression, from the foundational discoveries of Jinek et al. (2012), and Cong et al. (2013) on the CRISPR-Cas9 system to advancements in precision editing techniques by Gaudelli et al. (2017), and Anzalone et al. (2019).

Highly co-cited and cited works (Table 1) offer a framework for understanding the application of CRISPR-Cas9 in addressing age-related diseases. Foundational studies, including Ran et al. (2013) and Hsu et al. (2014), laid the groundwork for therapeutic innovations, driving significant progress in the use of genome editing technologies to combat aging-related conditions.

**TABLE 1:** Top ten highly co-cited and cited articles on CRISPR-Cas9 research in age-related diseases.

Top ten highly co-cited articles								
Rank	Author	Year	Journal	IF	JCR	Country	Co-cited	Title
1	Cong L	2013	Science	44.8	Q1	U.S.	18	Multiplex genome engineering using CRISPR/Cas systems (Cong et al., 2013)
2	Ran FA	2013	Nature Protocols	13.1	Q1	U.S.	18	Genome engineering using the CRISPR-Cas9 system (Ran et al., 2013)
3	Anzalone AV	2019	Nature	50.5	Q1	U.S.	16	Search-and-replace genome editing without double-strand breaks or donor DNA (Anzalone et al., 2019)
4	Hsu PD	2014	Cell	45.6	Q1	U.S.	14	Development and applications of CRISPR-Cas9 for genome engineering (Hsu et al., 2014)
5	Mali P	2013	Science	44.8	Q1	U.S.	12	RNA-guided human genome engineering via Cas9 (Mali et al., 2013)
6	Jinek M	2012	Science	44.8	Q1	U.S.	12	A programmable dual-RNA-guided DNA endonuclease in adaptive bacterial immunity (Jinek et al., 2012)
7	Gaudelli NM	2017	Nature	50.5	Q1	U.S.	11	Programmable base editing of A·T to G·C in genomic DNA without DNA cleavage (Gaudelli et al., 2017)
8	Labun K	2019	Nucleic Acids Research	16.7	Q1	Norway	10	CHOPCHOP v3: expanding the CRISPR web toolbox beyond genome editing (Labun et al., 2019)
9	Wang H	2013	Cell	45.6	Q1	U.S.	9	One-step generation of mice carrying mutations in multiple genes by CRISPR/Cas-mediated genome engineering (Wang et al., 2013)
10	Ran FA	2015	Nature	50.5	Q1	U.S.	9	*In vivo* genome editing using *Staphylococcus aureus* Cas9 (Ran et al., 2015)

Top ten highly cited articles								
Rank	Author	Year	Journal	IF	JCR	Country	Co-cited	Title
1	Marschallinger J	2020	Nature Neuroscience	21.3	Q1	U.S.	610	Lipid-droplet-accumulating microglia represent a dysfunctional and proinflammatory state in the aging brain (Marschallinger et al., 2020)
2	Soldner F	2011	Cell	45.6	Q1	U.S.	572	Generation of isogenic pluripotent stem cells differing exclusively at two early onset Parkinson point mutations (Soldner et al., 2011)
3	Banik SM	2020	Nature	50.5	Q1	U.S.	557	Lysosome-targeting chimaeras for degradation of extracellular proteins (Banik et al., 2020)
4	Zou Y	2020	Nature	50.5	Q1	China	475	Plasticity of ether lipids promotes ferroptosis susceptibility and evasion (Zou et al., 2020a)
5	Zou Y	2020	Nature Chemical Biology	13.0	Q1	China	465	Cytochrome P450 oxidoreductase contributes to phospholipid peroxidation in ferroptosis (Zou et al., 2020b)
6	Li HL	2015	Stem Cell Report	5.9	Q1	Japan	378	Precise correction of the dystrophin gene in Duchenne muscular dystrophy patient induced pluripotent stem cells by TALEN and CRISPR-Cas9 (Li et al., 2015)
7	Stroud DA	2016	Nature	50.5	Q1	Australia	373	Accessory subunits are integral for assembly and function of human mitochondrial complex I (Stroud et al., 2016)
8	Jain IH	2016	Science	44.8	Q1	U.S.	309	Hypoxia as a therapy for mitochondrial disease (Jain et al., 2016)
9	Pluvinage JV	2019	Nature	50.5	Q1	U.S.	285	CD22 blockade restores homeostatic microglial phagocytosis in ageing brains (Pluvinage et al., 2019)
10	Koblan LW	2021	Nature	50.5	Q1	U.S.	274	*In vivo* base editing rescues Hutchinson-Gilford progeria syndrome in mice (Koblan et al., 2021)

IF: Impact Factor (2024); JCR: Journal Citation Reports (2024).

### 3.7 Keyword clustering and bursting detection

**FIGURE 7:**
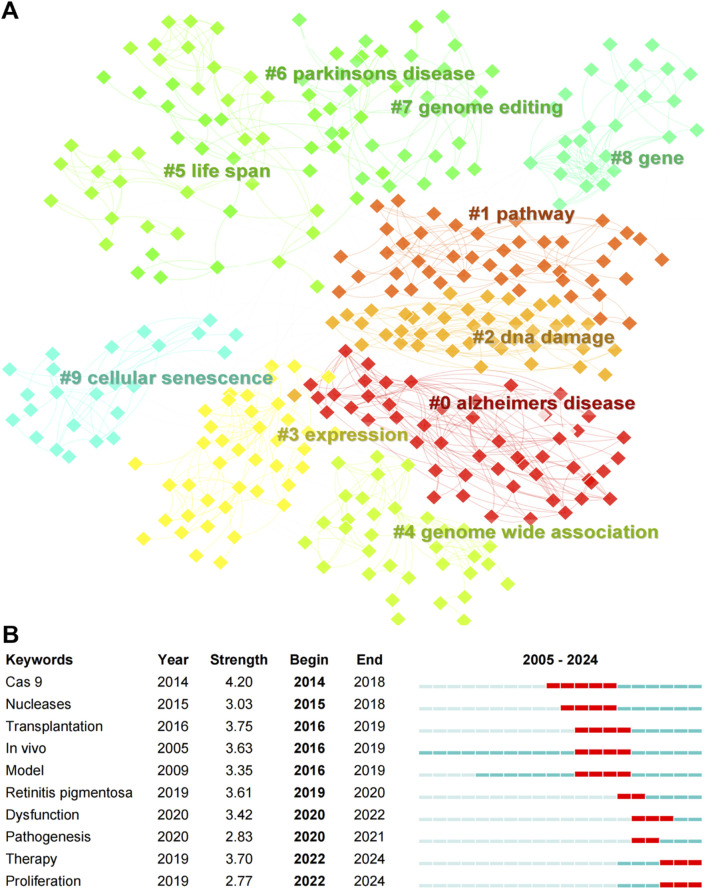
**(A)** Clustering map of keywords, and **(B)** top ten keywords with the strongest citation bursts (2005–2024) in CRISPR-Cas9 research on age-related diseases.

Keyword clustering analysis revealed the top ten clusters in CRISPR-Cas9 research on age-related diseases, including (0) Alzheimer’s disease, (1) Pathway, (2) DNA damage, (3) Expression, (4) Genome-wide association, (5) Life span, (6) Parkinson’s disease, (7) Genome editing, (8) Gene, and (9) Cellular senescence (Figure 7A). The modularity (Q) value of 0.8174 indicates a well-defined cluster structure (Q > 0.3), while the mean silhouette (S) value of 0.9004 confirms high reliability of the clustering (S > 0.7) (Yu and La, 2015). These clusters reflect the thematic focus and interdisciplinary nature of CRISPR-Cas9 applications in addressing key biological processes and diseases associated with aging. For instance, recent advances in Alzheimer’s disease (Cluster 0) highlight the translational potential of CRISPR-Cas9. Duan et al. utilized the blood-brain-barrier-penetrant AAV-PHP.eB vector to systemically deliver CRISPR-Cas9 in 5XFAD and *APP/PS1* transgenic mice, achieving allele-specific editing of the human *APPswe* gene (Duan et al., 2022). This intervention reduced Aβ pathology, mitigated microgliosis and neuritic dystrophy, and rescued cognitive deficits. Similarly, Park et al. demonstrated that amphiphilic R7L10 nanoparticle-mediated hippocampal delivery of CRISPR-Cas9 targeting *Bace1* in APPNL-G-F/NL-G-F and 5XFAD models attenuated BACE1 expression and ameliorated cognitive dysfunction5 (Park et al., 2019). These studies exemplify the growing emphasis on *in vivo* therapeutic strategies (Burst Keyword: *In vivo*, Strength 3.63) within Cluster 0. The Parkinson’s disease cluster (Cluster 6) further underscores innovations in delivery systems. Lipid nanoparticles (LNPs) encapsulating Cas9 protein and guide RNA (gRNA) have emerged as promising tools to protect CRISPR components from enzymatic degradation and enhance cellular uptake (Yadav et al., 2025). Surface functionalization of LNPs with targeting ligands, such as dopamine neuron-specific markers, enables precise delivery to degenerating neurons in Parkinson’s disease models (Wang et al., 2024). Polymer-based nanoparticles with tailored surface modifications similarly facilitate CRISPR/Cas9 targeting to dopamine pathways, illustrating the convergence of genome editing (Cluster 7) and translational neurotherapeutics.

Burst detection analysis identified the top ten keywords with the strongest citation bursts from 2005 to 2024 (Figure 7B). Among these, “Cas9” exhibited the highest burst strength (4.20) and the longest burst period, signifying its foundational role in the field. Other keywords with notable burst strengths include “Transplantation” (3.75) and “*In vivo*” (3.63), indicating their early significance in CRISPR-Cas9 research. The therapeutic focus is further emphasized by recent bursts in “Therapy” and “Dysfunction,” paralleling breakthroughs in retinal degeneration research. For example, Latella et al. demonstrated allele-specific disruption of the dominant *S334ter* mutation in the *RHO* gene via CRISPR/Cas9 in autosomal dominant retinitis pigmentosa (adRP) models (Latella et al., 2016). Subretinal delivery of gRNA/Cas9 plasmids prevented photoreceptor degeneration and restored visual function, validating its efficacy in gene editing (Cluster 7) for inherited retinal disorders (Cai et al., 2018). Such advances align with the rising prominence of therapy-oriented research (Burst Keyword: “Therapy”) in age-related disease models. These findings underscore the ongoing evolution of CRISPR-Cas9 research, with a shift toward translational and clinical advancements for combating age-related diseases.

## 4 Discussions

### 4.1 Summary of bibliometric analysis

This study reveals a global research landscape on CRISPR-Cas9 in age-related diseases, with the United States and China serving as central contributors, driven by institutions such as Harvard University and the Chinese Academy of Sciences. Keyword clustering and burst analyses highlight research hotspots in Alzheimer’s disease, Parkinson’s disease, gene editing, and cellular senescence, reflecting a transition from fundamental mechanisms to clinical applications. By identifying key contributors, major research focuses, and the phased characteristics of technological development, this study provides a comprehensive overview and valuable data to inform future research directions.

### 4.2 Insights from key findings

CRISPR-Cas9 has demonstrated significant therapeutic potential in age-related diseases, particularly in neurodegenerative disorders, metabolic diseases, and osteoporosis (Table 2). In Alzheimer’s disease, CRISPR-Cas9 reduces β-amyloid deposition by knocking out *APP* and *PSEN1* genes, alleviating neuroinflammation and improving cognitive function (Yoon et al., 2024). In Parkinson’s disease, it decreases α-synuclein aggregation by targeting the *SNCA* gene and restores mitochondrial function by correcting mutations in *LRRK2* and *PINK1* (Mansour and El-Khatib, 2023). Similarly, in cardiovascular diseases, targeting *PCSK9* lowers cholesterol levels, while *NLRP3* knockout reduces myocardial inflammation, improving atherosclerosis and decreasing cardiovascular event risk (Makhmudova et al., 2024). For osteoporosis, modulating the *SOST* gene enhances bone formation, while inhibiting *RANKL* reduces bone resorption, thereby increasing bone density and preventing fractures (Lin et al., 2024). These findings provide a foundation for direct molecular interventions in disease mechanisms. Recent research highlights the role of PANoptosis in immune infiltration during Alzheimer’s disease and the anti-aging effects of Panax notoginseng saponins through apoptosis and neurodegeneration pathways, offering novel therapeutic insights (Mei et al., 2024; Zhao et al., 2024).

**TABLE 2:** CRISPR-based therapeutic innovations for aging-related disorders: technological platforms, delivery systems, and preclinical models.

Aging-related disease	Application area	Research advances or examples	Mechanism or effect	CRISPR technology	Delivery method	Model organism/cell type
Alzheimer’s disease (Lu et al., 2021)	1. Correction of pathological gene mutations 2. Regulation of related gene expression	1. CRISPR/Cas9-mediated knockout of *APP* or *PSEN1* genes to reduce β-amyloid deposition 2. Targeting *ApoE4* to restore *ApoE3* function	Reduces amyloid plaque formation, lowers neuroinflammation, and improves cognitive function	*Staphylococcus aureus* Cas9	AAV9	*APP/PS1* transgenic mice Human iPSC-derived neurons
Parkinson’s disease (Maldonado et al., 2023)	1. Knockout of SNCA gene 2. Correction of gene mutations	1. Targeting *SNCA* to reduce α-synuclein aggregation 2. Repairing *LRRK2* or *PINK1* mutations to restore mitochondrial function	Prevents neurodegeneration and improves motor function	CRISPR-associated protein 12a	LNPs	*SNCA* triplication mice Primates and human dopaminergic neurons
Cardiovascular diseases (Makhmudova et al., 2024)	1. Regulation of lipid metabolism genes 2. Reduction of inflammatory responses	1. Targeting *PCSK9* to lower cholesterol levels 2. Knockout of *NLRP3 inflammasome* genes to reduce cardiac inflammation	Improves atherosclerosis and lowers the risk of cardiovascular events	Base editing	GalNAc-conjugated LNPs	ApoE^−/−^ mice Human iPSC-derived cardiomyocytes
Type 2 diabetes (Wal et al., 2024)	1. Correction of insulin resistance-related genes 2. Enhancement of insulin secretion	1. Targeting *INS* mutations to improve insulin secretion 2. Editing *PPARγ* or *FOXO1* to improve insulin sensitivity	Enhances glucose metabolism and delays diabetes-related complications	Prime editing	Electroporation of pancreatic β-cells	db/db mice Human islet organoids
Osteoporosis (Lin et al., 2024)	1. Regulation of bone formation 2. Reduction of bone resorption	1. Targeting *SOST* to enhance bone formation 2. Editing *RANKL* to suppress osteoclast activity	Increases bone density and prevents fractures	dCas9-VPR-mediated transcriptional activation	Hydrogel-encapsulated Cas9 mRNA	Ovariectomized rats hMSCs
Cancer (Wang et al., 2022)	1. Targeting aging-related oncogenic mutations 2. Regulation of telomerase activity	1. Targeting *p53*, *KRAS*, and other aging-associated mutations 2. Regulating *TERT* expression to maintain telomere length	Inhibits cancer cell proliferation and slows tumor progression	HiFi-Cas9	Tumor-targeted extracellular vesicles	PDX mice A549 lung cancer cells
Muscle degenerative diseases (Li et al., 2015)	1. Correction of gene mutations 2. Enhancement of muscle regeneration	1. CRISPR-mediated correction of mutations to treat Duchenne muscular dystrophy 2. Targeting *MSTN* to promote muscle growth	Enhances muscle strength and mitigates aging-associated muscle loss	Dual-sgRNA Cas9 system	AAVrh74	mdx mice Human myoblasts
Retinal degenerative diseases (Peng et al., 2017)	1. Correction of retinal gene mutations 2. Mitigation of vision deterioration	1. Targeting *CEP290* mutations to treat Leber congenital amaurosis 2. Editing *VEGF* to reduce vascular abnormalities in age-related macular degeneration	Restores visual function and slows retinal degeneration	Mini-Cas9	Subretinal injection of AAV5	Rd10 mice Human retinal pigment epithelial organoids
Aging (Caobi et al., 2020)	1. Targeting key aging-related genes 2. Enhancing cellular regeneration	1. Targeting *p16* and *p21* to delay cellular senescence 2. Editing mitochondrial-related genes (e.g., *TFAM*) to improve mitochondrial function	Delays tissue degeneration and promotes organ function restoration	Cas9-cytidine deaminase fusion	LNPs with ionizable lipids	Senescence-accelerated mice Human fibroblasts

AAV: Adeno-associated virus; AAVrh74: Adeno-associated virus serotype rh74; APP: amyloid precursor protein; ApoE4/ApoE3: Apolipoprotein E; Cas12a: CRISPR-associated protein 12a; CBE: Cas9-cytidine deaminase fusion; CEP290: Centrosomal Protein 290; dCas9-VPR: Deactivated Cas9 fused to VP64-p65-Rta tripartite activation domain; FOXO1: Forkhead Box Protein O1; GalNAc: N-Acetylgalactosamine; HiFi-Cas9: High-Fidelity Cas9; hMSCs: Human mesenchymal stem cells; INS: insulin; iPSC: induced pluripotent stem cell; LRRK2: Leucine-Rich Repeat Kinase 2; LNPs: Lipid nanoparticles; MSTN: myostatin; NLRP3: NOD-, LRR-, and Pyrin Domain-Containing Protein 3; PCSK9: Proprotein Convertase Subtilisin/Kexin Type 9; PINK1: PTEN-Induced Kinase 1; PPARγ: Peroxisome Proliferator-Activated Receptor Gamma; PDX: Patient-derived xenograft; PSEN1: Presenilin 1; RANKL: Receptor Activator of Nuclear Factor Kappa-B, ligand; RPE: retinal pigment epithelial; SaCas9: *Staphylococcus aureus* Cas9; SOST: sclerostin; TERT: telomerase reverse transcriptase; TFAM: Transcription Factor A, mitochondrial; VEGF: vascular endothelial growth factor.

On the technical front, high-fidelity (HiFi) Cas9 variants, such as eSpCas9 and HiFi Cas9, have significantly minimized off-target effects (Chen et al., 2017; Vakulskas et al., 2018), while HDR-independent tools like base editing and prime editing have improved gene editing efficiency in non-dividing cells (Kantor et al., 2020). Advances in non-viral delivery systems and low-immunogenicity Cas9 variants address challenges of delivery inefficiency and high immune risks (Li et al., 2018; Kenjo et al., 2021). These innovations enhance the precision, safety, and feasibility of CRISPR-Cas9 in both research and potential clinical applications for age-related diseases.

### 4.3 Challenges and barriers to clinical translation

Co-citation analyses and preclinical validation studies consistently identify off-target effects as the primary technical constraint limiting clinical translation of CRISPR-Cas9 systems (Yuan, 2025). Emerging data demonstrate that nonspecific Cas9 activity not only induces genomic instability but also disrupts epigenetic regulatory networks, presenting particular risks in polygenic age-related disorders where pathway interdependencies magnify biological consequences (Table 3). These challenges manifest most prominently within two interconnected research domains: DNA damage repair mechanisms associated with error-prone nonhomologous end joining, and gene expression dysregulation stemming from unintended editing of regulatory elements. Technological innovations are addressing these concerns, as evidenced by high-fidelity editors such as HiFi-Cas9 achieving 20-fold reductions in off-target activity within human hematopoietic stem cells (Vakulskas et al., 2018). Concurrently, artificial intelligence platforms exemplified by DeepCRISPR provide robust prediction of guide RNA specificity, attaining 0.981 ROC-AUC values in cross-validated datasets (Chuai et al., 2018). However, maintaining functional equilibrium in aging-related gene networks requires further refinement of these precision tools, particularly when targeting genomic regions containing homologous pseudogenes or repetitive sequences.

**TABLE 3:** Key challenges and advanced solutions in CRISPR/Cas9 genome editing.

Challenges	Specific issues	Proposed solutions or improvements	Evidence-based examples
Off-target effects	Unintended edits at non-target genomic sites, leading to mutations or functional disruptions	• Development of high-fidelity Cas9 variants • AI-guided sgRNA design	• HiFi-Cas9 reduced off-target activity by 20-fold in human hematopoietic stem cells (Vakulskas et al., 2018) • DeepCRISPR achieves an ROC-AUC of 0.981 for predicting off-target sites (Chuai et al., 2018)
Low editing efficiency	Inefficient editing in non-dividing cells or heterochromatin regions	• Engineered Cas9 variants with enhanced nuclear localization signals • Chromatin-modulating adjuvants to relax chromatin accessibility	• xCas9 demonstrates higher editing efficiency than SpCas9 for diverse PAM sequences, including NG, GAA, and GAT. (Hu et al., 2018) • HDAC inhibitors increased HDR and NHEJ efficiencies by 2.8 - fold and 1.5 - fold respectively (Zhang et al., 2021)
Delivery challenges	Difficulty in efficient delivery to specific tissues or cell types	• Viral vector optimization • Hybrid delivery systems	• AAV delivery of a miniaturized dystrophin gene improves symptoms in the Golden Retriever muscular dystrophy model without causing severe adverse events (Birch et al., 2023) • Targeted delivery of αEGFR-CRISPR-sgSOX2-LNPs resulted in a 90% inhibition of tumor growth and a 90% increase in survival for over 84 days in a xenograft HNSCC mouse model (Masarwy et al., 2025)
Immunogenicity risks	Host immune responses triggered by bacterial-derived Cas9 or delivery vehicles	• Identification and modification of B and T cell epitopes in Cas9 proteins • Stealth LNPs coated with CD47 to evade macrophage clearance	• Mutation of the immunodominant B cell epitope (R338) in SaCas9 reduces antibody binding without losing cleavage function (Shen et al., 2022) • CD47 functionalized NPs have significantly reduced macrophage uptake and a longer circulation time in the body (Gheibi Hayat et al., 2019)
HDR dependency	HDR inefficiency in non-dividing cells limits therapeutic applications	• Developing HDR-independent editors	• PE6 variants achieved a 24-fold improvement in loxP insertion following dual-AAV delivery compared to previous PE systems (Doman et al., 2023)
Ethical and regulatory concerns	Germline editing and potential misuse raise significant ethical challenges	• Strengthening ethical regulatory frameworks; restricting the scope of technology application	• The condemnatory statement, urging stance, and propositional viewpoints of the Chinese Academy of Sciences on the gene-edited baby incident (Wang et al., 2019; Wang and Yang, 2019)

AAV: Adeno-Associated Virus; Cas9: CRISPR-associated protein 9; eSpCas9: Enhanced Specificity Cas9; HDR: Homology-Directed Repair; HiFi Cas9: High Fidelity Cas9; LNPs: Lipid Nanoparticles; NHEJ: Non-Homologous End Joining; sgRNA: Single Guide RNA; xCas9: Engineered Cas9 variant with expanded PAM, compatibility.

Delivery limitations, prominently identified through citation burst dynamics of keywords such as “Transplantation” and “*In vivo*,” persist as critical translational barriers. Current strategies increasingly adopt multifunctional integration paradigms to simultaneously address tissue selectivity, delivery efficiency, and immune compatibility. Viral vector optimization efforts demonstrate particular promise, exemplified by AAV-mediated delivery of miniaturized dystrophin constructs achieving functional recovery in canine muscular dystrophy models with negligible adverse events (Birch et al., 2023). Conversely, non-viral approaches are advancing through ligand-directed targeting, as evidenced by epidermal growth factor receptor functionalized lipid nanoparticles delivering CRISPR-SOX2 therapeutics that achieved 90% tumor growth suppression and extended survival beyond 84 days in head-and-neck squamous cell carcinoma xenografts (Masarwy et al., 2025). These case studies underscore the potential of hybrid delivery architectures combining viral precision with non-viral safety profiles. Nevertheless, critical gaps remain: AAV systems face payload capacity limitations for larger Cas9 variants, while nanoparticle formulations exhibit suboptimal penetration through fibrotic microenvironments characteristic of aged tissues.

Ethical considerations and longitudinal safety uncertainties remain inextricably linked to CRISPR-Cas9 clinical development. Germline editing controversies, particularly regarding irreversible hereditary modifications and transgenerational epigenetic effects, have catalyzed international scientific consensus-building efforts. The 2018 gene-edited infant incident precipitated rigorous institutional responses, including the Chinese Academy of Sciences’ seminal position statement advocating for stringent oversight frameworks and restricted therapeutic applications (Wang et al., 2019).

### 4.4 Future directions and recommendations

To address current technical and ethical challenges, the future development of CRISPR-Cas9 should focus on optimizing precision, enhancing delivery strategies, and establishing robust regulatory frameworks. For off-target effects, high-precision sgRNA design platforms and single-base editors offer improved specificity and safety in polygenic environments (Doench et al., 2016; Hryhorowicz et al., 2023). Tools independent of HDR, such as prime editors, could further expand applicability by overcoming low editing efficiency in non-dividing cells (Kantor et al., 2020). In terms of delivery, ligand-modified lipid nanoparticles show promise for improving tissue specificity, while combining viral and non-viral delivery methods could enhance efficiency and mitigate immunogenic risks (Li et al., 2018). To address long-term safety concerns, developing low-immunogenic Cas9 variants and incorporating controllable gene-editing switches could enable reversible and precise interventions.

On the ethical and regulatory front, dynamic ethical oversight and international collaboration are essential for establishing unified frameworks to govern the use of germline editing. Multicenter clinical trial networks should prioritize gathering long-term safety and efficacy data to ensure the responsible application of CRISPR-Cas9. These strategies aim to seamlessly bridge technological advancements and clinical translation, accelerating its application in treating age-related diseases.

### 4.5 Limitations

This study has certain limitations, primarily in the scope of data coverage and the depth of technical analysis. While we retrieved a substantial number of publications from major databases such as Web of Science, the exclusion of regional studies and non-English literature may result in incomplete global coverage. To minimize this bias, we employed a multi-database integration approach and a rigorous three-reviewer screening process. However, future studies should incorporate regional databases (e.g., CNKI) and open-access resources (e.g., bioRxiv) to enhance data comprehensiveness. Additionally, although this study systematically identified the technical challenges of CRISPR-Cas9, it provides limited exploration of the molecular mechanisms underlying these issues and cutting-edge solutions. Future work should integrate experimental data and disease-specific models to validate the biological efficacy of proposed strategies, offering more concrete guidance for advancing the technology.

## 5 Conclusion

This study provides a comprehensive overview of the global development, therapeutic potential, and technical challenges of CRISPR-Cas9 in age-related disease research. The findings highlight its promising applications in gene repair and functional regulation for diseases such as Alzheimer’s and Parkinson’s. However, significant barriers remain, including off-target effects, low delivery efficiency, and challenges in editing non-dividing cells, alongside ethical concerns over germline editing and long-term safety. Future efforts should prioritize the development of high-precision Cas9 variants, HDR-independent tools, and efficient delivery systems. Integrating multicenter clinical trials with an international ethical framework will be critical to unlocking the transformative potential of CRISPR-Cas9 for treating age-related diseases.

## Data Availability

The original contributions presented in the study are included in the article/Supplementary Material, further inquiries can be directed to the corresponding authors.

## Abbreviations

APP, amyloid precursor protein; CNKI, China National Knowledge Infrastructure; CRISPR-Cas9, clustered regularly interspaced short palindromic repeats-associated protein 9; eSpCas9, enhanced specificity Cas9; HDR, homology-directed repair; HiFi Cas9, high-fidelity Cas9; LRRK2, leucine-rich repeat kinase 2; NHEJ, non-homologous end joining; NLRP3, NOD-, LRR-, and pyrin domain-containing protein 3; PCSK9, proprotein convertase subtilisin/kexin type 9; PINK1, PTEN-induced kinase 1; PSEN1, presenilin 1; RANKL, receptor activator of nuclear factor kappa-Β ligand; sgRNA, single-guide RNA; SNCA, synuclein alpha.
