# Supplementary material for: Mapping the therapeutic landscape of CRISPR-Cas9 for combating age-related diseases
Source: Front Genome Ed. 2025 Apr 4;7:1558432. doi: 10.3389/fgeed.2025.1558432 (PMC12006052; doi:10.3389/fgeed.2025.1558432)
Supplement: Supplementary file 1 [file Supplementaryfile1.docx]

***Supplementary Material***

**Table S1** Detailed search strategies and outcomes.

| Database | Query | Results |
| --- | --- | --- |
| **PubMed** | #1: (CRISPR-Cas9[Title/Abstract]) OR (Genome Editing[Title/Abstract]) OR (Gene Editing[Title/Abstract]) OR (CRISPR Technology[Title/Abstract]) OR (CRISPR Therapeutics[Title/Abstract]) OR (Clustered Regularly Interspersed Short Palindromic Repeats[Title/Abstract]) OR (Cas9[Title/Abstract]) | 45,622 |
|  | #2: (Age-Related Diseases) OR (Aging-Associated Disorders[Title/Abstract]) OR (Aging-Related Diseases[Title/Abstract]) OR (Age-Associated Diseases[Title/Abstract]) OR (Aging Disorders[Title/Abstract]) OR (Diseases of Aging[Title/Abstract]) OR (Degenerative Diseases[Title/Abstract]) OR (Anti-Aging[Title/Abstract]) OR (Longevity[Title/Abstract]) OR (Healthy Aging[Title/Abstract]) OR (Cellular Senescence[Title/Abstract]) | 134,002 |
|  | #3: #1 AND #2 | **371** |
| **Embase** | #1: 'CRISPR-Cas9':ab,ti OR 'Genome Editing':ab,ti OR 'Gene Editing':ab,ti OR 'CRISPR Technology':ab,ti OR 'CRISPR Therapeutics':ab,ti OR 'Clustered Regularly Interspersed Short Palindromic Repeats':ab,ti OR 'Cas9':ab,ti | 61,570 |
|  | #2: 'Age-Related Diseases':ab,ti OR 'Aging-Associated Disorders':ab,ti OR 'Aging-Related Diseases':ab,ti OR 'Age-Associated Diseases':ab,ti OR 'Aging Disorders':ab,ti OR 'Diseases of Aging':ab,ti OR 'Degenerative Diseases':ab,ti OR 'Anti-Aging':ab,ti OR 'Longevity':ab,ti OR 'Healthy Aging':ab,ti OR 'Cellular Senescence':ab,ti | 10,295 |
|  | #3: #1 AND #2 | **366** |
| **Cochrane** | #1: (CRISPR-Cas9):ab,ti,kw OR (Genome Editing):ab,ti,kw OR (Gene Editing):ab,ti,kw OR (CRISPR Technology):ab,ti,kw OR (CRISPR Therapeutics):ab,ti,kw OR (Clustered Regularly Interspersed Short Palindromic Repeats):ab,ti,kw OR (Cas9):ab,ti,kw | 130 |
|  | #2: (Age-Related Diseases):ab,ti,kw OR (Aging-Associated Disorders):ab,ti,kw OR (Aging-Related Diseases):ab,ti,kw OR (Age-Associated Diseases):ab,ti,kw OR (Aging Disorders):ab,ti,kw OR (Diseases of Aging):ab,ti,kw OR (Degenerative Diseases):ab,ti,kw OR (Anti-Aging):ab,ti,kw OR (Longevity):ab,ti,kw OR (Healthy Aging):ab,ti,kw OR (Cellular Senescence):ab,ti,kw | 11,561 |
|  | #3: #1 AND #2 | **3** |
| **Scopus** | #1: TITLE-ABS-KEY (“CRISPR-Cas9” OR “Genome Editing” OR “Gene Editing” OR “CRISPR Technology” OR “CRISPR Therapeutics” OR “Clustered Regularly Interspersed Short Palindromic Repeats” OR “Cas9”) | 76,490 |
|  | #2: TITLE-ABS-KEY (“Age-Related Diseases” OR “Aging-Associated Disorders” OR “Aging-Related Diseases” OR “Age-Associated Diseases” OR “Aging Disorders” OR “Diseases of Aging” OR “Degenerative Diseases” OR “Anti-Aging” OR “Longevity” OR “Healthy Aging” OR “Cellular Senescence”) | 250,207 |
|  | #3: #1 AND #2 | **1,218** |
| **Web of science** | #1: TS = (CRISPR-Cas9 OR Genome Editing OR Gene Editing OR CRISPR Technology OR CRISPR Therapeutics OR Clustered Regularly Interspersed Short Palindromic Repeats OR Cas9) | 63,040 |
|  | #2: TS = (Age-Related Diseases OR Aging-Associated Disorders OR Aging-Related Diseases OR Age-Associated Diseases OR Aging Disorders OR Diseases of Aging OR Degenerative Diseases OR Anti-Aging OR Longevity OR Healthy Aging OR Cellular Senescence) | 1,708,750 |
|  | #3: #1 AND #2 | **1,267** |

**Table S2** Top ten authors contributing to CRISPR-Cas9 research in age-related diseases.

| **Rank** | **Authors** | **No. of publications** | **Institutions** | **Countries** |
| --- | --- | --- | --- | --- |
| 1 | Liu GH | 15 | Chinese Academy of Sciences | China |
| 2 | Qu J | 14 | Chinese Academy of Sciences | China |
| 3 | Zhao W | 11 | Chinese Academy of Sciences | China |
| 4 | Belmonte JCI | 9 | Salk Institute for Biological Studies | U.S. |
| 5 | Liu Z | 7 | Chinese Academy of Sciences | China |
| 6 | Zhang Yu | 9 | Tianjin Medical University | China |
| 7 | Brunet, Anne | 5 | Stanford University | U.S. |
| 8 | Wang S | 8 | Chinese Academy of Sciences | China |
| 9 | Song M | 5 | Chinese Academy of Sciences | China |
| 10 | Yang Y | 4 | Shanghai Jiao Tong University | China |

**Table S3** Top ten institutions publishing research on CRISPR-Cas9 and age-related diseases.

| **Rank** | **Institutions** | **No. of publications** | **Countries** |
| --- | --- | --- | --- |
| 1 | Harvard University | 87 | U.S. |
| 2 | Chinese Academy of Sciences | 77 | China |
| 3 | University of California system | 45 | U.S. |
| 4 | Institut National de la Santé et de la Recherche Médicale | 32 | France |
| 5 | University of London | 32 | U.K. |
| 6 | Centre national de la recherche scientifique | 27 | France |
| 7 | National Institutes of Health | 26 | U.S. |
| 8 | University of Texas system | 26 | U.S. |
| 9 | Peking University | 23 | China |
| 10 | University system of Ohio | 22 | U.S. |

**Table S4** Top ten funding agencies supporting CRISPR-Cas9 research for combating age-related diseases.

| **Rank** | **Funding agencies** | **No. of publications** | **Countries or regions** |
| --- | --- | --- | --- |
| 1 | National Institutes of Health | 280 | U.S. |
| 2 | U.S. Department of Health and Human Services | 280 | U.S. |
| 3 | National Natural Science Foundation of China | 151 | China |
| 4 | Japan Society for the Promotion of Science | 35 | Japan |
| 5 | National key Research Development Program of China | 35 | China |
| 6 | Grants-in-Aid for Scientific Research | 33 | Japan |
| 7 | German Research Foundation | 32 | Germany |
| 8 | UK Research and Innovation | 30 | U.K. |
| 9 | European Research Council | 25 | European Union |
| 10 | Wellcome Trust | 24 | U.K. |

**Table S5** Top ten journals publishing on CRISPR-Cas9 research in age-related diseases.

| **Rank** | **Journals** | **IF** | **JCR** | **No. of publications** |
| --- | --- | --- | --- | --- |
| 1 | International Journal of Molecular Sciences | 4.9 | Q1 | 33 |
| 2 | Scientific Reports | 3.8 | Q1 | 25 |
| 3 | Nature Communications | 14.7 | Q1 | 19 |
| 4 | Human Molecular Genetics | 3.1 | Q2 | 18 |
| 5 | Proceedings of the National Academy of Sciences of the United States of America | 9.4 | Q1 | 16 |
| 6 | Cell Death Disease | 8.1 | Q1 | 15 |
| 7 | Investigative Ophthalmology & Visual Science | 5.0 | Q1 | 14 |
| 8 | Aging Cell | 8.0 | Q1 | 11 |
| 9 | eLife | 6.4 | Q1 | 10 |
| 10 | Nature | 50.5 | Q1 | 9 |

IF: Impact Factor (2024); JCR: Journal Citation Reports (2024).
